# Systematic review of risk factors for violence in psychosis: a 10-year update

**DOI:** 10.1192/bjp.2024.120

**Published:** 2025-03-17

**Authors:** Tyra Lagerberg, Sinéad Lambe, Anabelle Paulino, Rongqin Yu, Seena Fazel

**Affiliations:** 1 Department of Psychiatry, Warneford Hospital, University of Oxford, Oxford, UK; 2 Department of Medical Epidemiology and Biostatistics, Karolinska Institutet, Stockholm, Sweden; 3 Oxford Health NHS Foundation Trust, Oxford, UK

## Abstract

**Background:**

Understanding risk factors for violence in people with psychosis can inform risk management and violence prevention. However, much of the evidence comes from cross-sectional studies, and previous reviews require updating.

**Aims:**

To synthesize evidence from longitudinal studies on risk factors for violence in people with schizophrenia-spectrum disorders, bipolar disorder, or other affective psychoses.

**Method:**

We searched five bibliographic databases up to June 2022. We identified longitudinal studies reporting risk factors for violence in individuals diagnosed with schizophrenia or other psychoses using DSM or ICD criteria. If ≥3 independent samples reported a risk factor, we conducted random-effects meta-analyses to provide a pooled estimate. We also meta-analysed risk factors by major domains.

**Results:**

We identified 47 longitudinal studies on risk factors for violence in psychosis, representing 41 independent samples – 21 from the original and 20 from the updated review– and 203,297 individuals. 30 risk factors were present in ≥3 independent samples. Criminal history factors were associated with the greatest risk of violent crime (pooled OR=3.50, 95%CI=2.37, 5.16), followed by substance misuse factors (OR=2.36, 95%CI=1.99, 2.80). Many treatment-related factors were protective (OR=0.54, 95%CI=0.34, 0.85). Effect estimates were attenuated in inpatient settings. We also identified novel risk factors, including cannabis misuse, in a secondary analysis (OR=3.34, 95%CI=2.32, 4.82).

**Conclusions:**

Using longitudinal evidence, we have validated comorbid substance misuse and criminal history as major risk factors for violence in psychosis. Novel factors such as cannabis misuse need further replication. Several identified factors are possible intervention targets if associations are found to be causal.

## Introduction

Given the high costs of violence perpetration to patients, victims, and society,^1 2^ preventing violent outcomes and improving risk management is a priority for clinical services.^3^ Triangulated evidence show a higher risk of violence among individuals with psychotic disorders than among those without.^4–6^ Absolute risks of violence can be as high as 26% over 12 months in first episode psychosis,^7^ but are more typically less than 10% within 5 years of diagnosis by clinical services.^5^ Identification of risk factors – particularly modifiable ones – in people with psychotic disorders is a next step in developing targeted interventions,^8^ and could help develop more precise risk assessment tools that allow for risk stratification. Such tools are common in forensic mental health and criminal justice to aid clinical decision-making, but of varying accuracy.^9^ Updated evidence on risk factors is particularly relevant for treatment allocation in the context of limited resources, for example with the reduction of available psychiatric hospital beds in the UK and US that has continued in recent years.^10^

A 2013 systematic review^11^ of risk factors for violence in psychosis outlined a range of replicated risk markers for criminal history, psychopathological symptoms, and treatment-related factors. However, the previous review is now more than a decade old with its search ending in 2011, and many new investigations have since been published. Furthermore, the previous review included a majority of cross-sectional studies where the temporal relationship between the studied factor and violent outcome is not clear. In this update, we have focused on longitudinal studies to improve the quality of the evidence. In addition, we have conducted separate analyses restricting studies to those using more severe violent outcomes, and those where a majority of participants were recruited from inpatient settings. The latter can inform how to prevent and manage inpatient violence, which has been reported to occur in 21-32% of hospitalized individuals with psychotic disorders.^12^ We consider risk factors in a broad way to include descriptive, causal, and predictive associations.^13^ We anticipated replicating the strongest risk factors in the previous review – including comorbid substance use disorders and criminal history – while drawing on a decade of new evidence on emerging risk factors for violence in psychosis, including the misuse of individual substances.

## Methods

The study was conducted following the Preferred Reporting Items for Systematic Reviews and Meta-Analyses (PRISMA) guidelines.

### Protocol

The review methods are based on a previous review from our group,^11^ published open access in 2013. One deviation from this protocol was to limit the inclusion to longitudinal designs, which were included in the original protocol alongside cross-sectional designs. We further excluded studies in selected (e.g. solely offender) populations. There were no other material deviations from the original protocol.

### Search strategy

We implemented the same search strategy as the 2013 review,^11^ using the following search term to identify studies examining psychiatric disorders and various violence measures: (schiz* AND (viol* OR aggress* OR crim* OR offend* OR danger* OR hosti*)) OR (psych* AND (viol* OR aggress* OR crim* OR offend* OR danger* OR hosti*)) OR (mental* AND (viol* OR aggress* OR crim* OR offend* OR danger* OR hosti*)) (Table S1). We conducted the search in five databases – CINAHL, Embase, Global Health, PsycINFO and PubMed – to identify papers published from 1^st^ January 2012 until 30^th^ June 2022. We thus complemented the search from the original review, which searched evidence until 31^st^ December 2011. The extraction of references from the databases was carried out on 30^th^ June 2022. We also conducted a manual search of reference lists in included or related papers. We translated non-English language publications using Google Translate and asked a native speaker for clarification where necessary. Search and eligibility assessment was carried out by the first author (TL). A second reviewer was not considered for systematic eligibility assessment, in line with the previous review.

### Study eligibility

We considered studies where: (1) at least 95% of the sample were diagnosed with schizophrenia, schizoaffective disorder, delusional disorder, other schizophrenia-spectrum disorder, schizotypal disorder, bipolar disorder, or other affective psychosis, excluding drug-induced psychosis (e.g. if a study included 94% patients with schizophrenia spectrum disorder and 6% with drug-induced psychosis or a non-psychotic disorder then that study would be excluded); (2) diagnosis was made using Diagnostic and Statistical Manual of Mental Disorders (DSM) or International Classification of Diseases (ICD) criteria; (3) individuals were aged 15 years or above; and (4) the study employed a design where risk factors preceded the outcome (e.g. cohort studies, nested case-control, RCTs, and prediction studies). Further, we (5) excluded studies that only considered repeat violence as an outcome – that is, any study examining violent outcomes in a selected population with violence histories. As in the previous review, bipolar disorder was included as psychosis is a feature in most individuals with bipolar disorder,^14^ and to incorporate risk factors for violence in affective psychosis. For each study considered for inclusion, we extracted information on each inclusion criterion 1-5 – the first inclusion criterion we noticed that was not fulfilled was logged as the reason for that paper’s exclusion.

### Data extraction

Data from included papers was extracted by the first author (TL). A second author (SL) independently extracted a randomly selected 20% subset of included studies to assess extraction accuracy. For both risk factors and violent outcomes, we extracted information on whether the variable was categorical or continuous. Risk factor definitions across studies were standardized where possible to ensure they could be pooled. In keeping with previous work,^11^ we classified risk factors into the following broad and distinctive domains: criminal history, negative symptoms, neuropsychological, positive symptoms, premorbid, psychopathological, sociodemographic, substance misuse, suicidality, and treatment related.

To ensure comparability with previous work and allow for data pooling, we converted all effect estimates to odds ratios (ORs), using methods described in previous publications.^11 15^ Hazard ratios (HRs) and probit regression coefficients cannot strictly be converted to ORs. However, probit and logistic regression give very similar results, and HRs can be relatively similar to ORs if the outcome event is rare by the end of follow-up.^16^ We therefore included these effect estimates in the main analysis. As a sensitivity analysis, we excluded all effect estimates measured by HRs or probit regression coefficients.

Study quality was assessed by co-author AP using an adaptation of the Newcastle-Ottawa Scale^17^ where we scored quality of exposure ascertainment separately for each risk factor domain included in the study. The overall quality score was then produced by summing the quality score for each risk factor domain included with the other sub-scores (selection, comparability, and outcome). The score was expressed as a percentage of the maximum quality score available given the risk factor domains included in the study.

### Statistical analyses

We assessed extraction accuracy by calculating inter-rater reliability for the extraction of the proportion of violent patients using Krippendorff’s alpha.^18^

We pooled ORs using random effects models due to the heterogeneity in the design and predictors/outcomes of studies. We only considered risk factors that occurred in at least three independent samples in the main analysis, but reported results for risk factors occurring in two samples in the supplement. We chose to pool the minimally adjusted effect estimate available, as this was the most consistently reported and comparable.^11 15^ Some included papers were based on the same original study. If a risk factor occurred in more than one paper that used the same study population, we included the risk factor that derived from the paper with the largest sample size to avoid double counting.^11^ All analyses in the current paper were carried out at the level of independent samples rather than publications.

We additionally pooled ORs within each risk factor domain. If one paper contributed more than one risk factor per domain, we included the risk factor with the highest absolute z-score. The z-score takes into account the size of the effect estimate (strength of association) and its standard deviation (precision).^11^

We assessed heterogeneity using the I^2^ statistic to quantify the proportion of the variance in the risk factor effect estimates that are due to between-study differences rather than random sampling error. To investigate the sources of between-study variability, we ran meta-regression models for risk factors that occurred in at least 7 samples and had an I^2^ of ≥75%. We considered the following between-study factors in the meta-regressions: whether the study setting was in Europe or not (binary), whether a majority of study subjects were recruited from an inpatient setting or not (binary), percentage of men (continuous), and whether the violent outcome was based on forensic care status/criminal record or not (binary).

### Sensitivity analyses

To account for different settings and violent outcome types across samples, we conducted analyses including risk factors from only: a) samples where violence was defined by conviction/arrest (violence and/or homicide) or forensic psychiatric care (which typically requires a criminal offence); or b) samples where 95% or more of the population was recruited from inpatient settings. We also conducted analyses where we excluded risk factors with effect estimates reported as HRs and probit regression coefficients. We further restricted analyses to those papers that were deemed to have a quality score of ≥75%. Finally, we conducted publication bias analyses for risk factors that occurred in at least 7 independent samples using Peters’ regression test.^19^

Data management and analyses, including effect estimate conversions, were carried out in R version 4.3.0.

## Results

### Study characteristics

We identified 79,988 publications from the five listed databases after removal of duplicates; 288 remained after a title and abstract screening. After full-text screening, 47 studies were included in this update, representing 41 independent samples (Figure S1, Table S3). ^5 20–65^ Four studies stratified their findings by sex and one further study was stratified by diagnostic category (schizophrenia vs bipolar disorder), and thus each contributed two independent sets of risk factors to analyses.^5 33 50 58 59^ Fourteen studies overall were overlapping, some of which incorporated aforementioned sex- or diagnosis-stratified samples.^21–23 25 33–35 51 56 58–60 62 63^ Overall, 564 separate effect sizes were extracted, representing 387 unique risk factors after standardization. Of these, 30 were examined in at least three independent samples, and an additional 30 factors occurred in two. The publication years ranged from 1983 to 2022, with the majority of samples originating from Europe (k=20, 49%), followed by the USA (k=10) and the UK (k=5). Median sample size was 404 (Table 1). Ten samples (24%) recruited more than 95% of their participants from inpatient settings. The majority measured their violent outcome as a physical assault on another person (k=12, 29%) or as a conviction for a violent crime (k=12, 29%; Table 1).

We found good interrater reliability of the extraction of the proportion of violent individuals (Krippendorff’s alpha=0.77).

**Figure 1:**
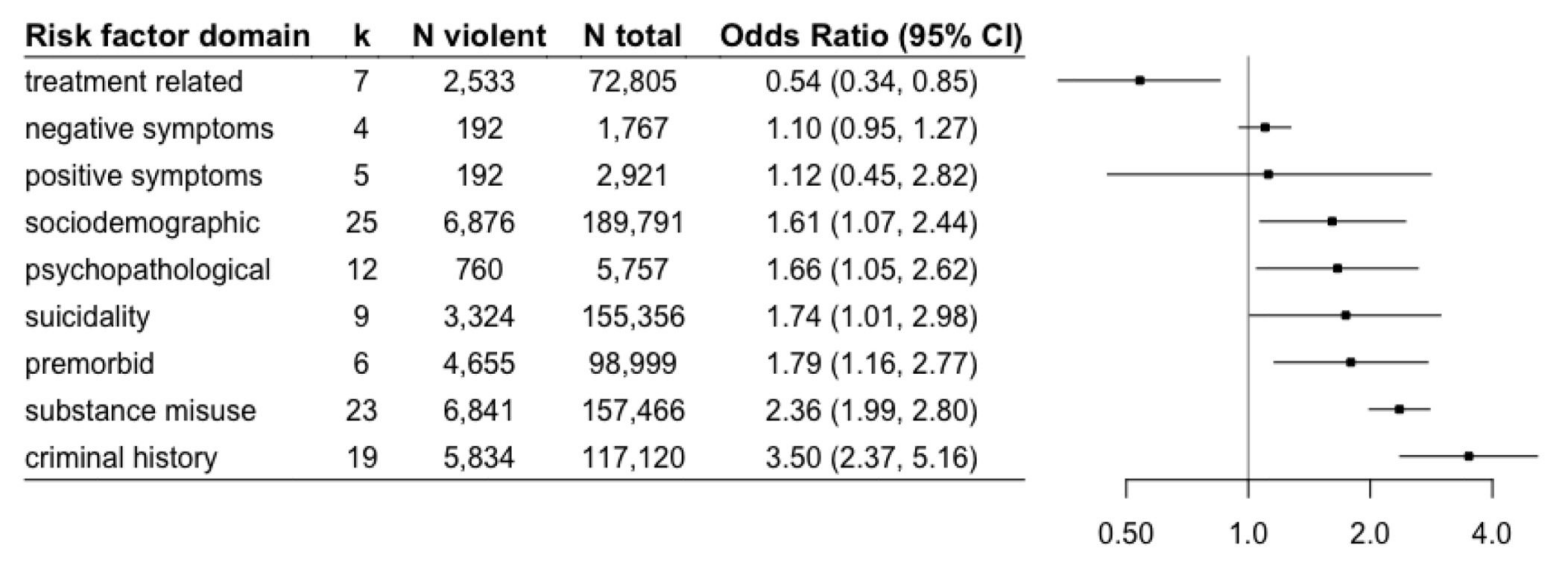
Pooled odds ratios for violence in psychosis by overall risk factor domain

Table 2 shows the effect estimates of risk factors that were reported in ≥3 independent samples, pooled over the individual risk factors. Figure 1 illustrates the effect estimates when these risk factors were pooled over risk factor domains. No risk factors in the neuropsychological domain were present in two or more samples, and hence no factors from the neuropsychological domain were included in the analyses. We allowed individual PANSS items to be included as separate risk factors.

### Criminal history domain

OR estimates were similar across criminal history factors (Table 2), and were all associated with an increased risk of violence in people with psychosis. The most commonly reported criminal history risk factor was “violence history” (k=15), with an OR of 2.91 (95%CI=2.06, 4.10). After pooling across all criminal history factors, the overall domain was associated with more than a three-fold increased risk (OR=3.50, 95%CI=2.37, 5.16; Figure 1). A history of non-violent crime and a family history of offending behaviour were also risk factors and reported in two independent samples (Table S4).

### Negative and positive symptom domains

Risk factors present in three or more independent samples were the negative symptom score on the Positive and Negative Syndrome Scale (PANSS) (OR=1.10, 95%CI=0.95, 1.27) and positive symptom score on the PANSS (OR=1.12, 95%CI=0.45, 2.82) (Table 2), though neither showed a clear association with violence. In the positive symptom domain, paranoia and hostility were reported in two independent samples (Table S4), with paranoia showing the greatest point estimate (OR=3.07, 95%CI=0.88, 10.69).

### Premorbid domain

Two premorbid factors were associated with an increased risk of violence: recent violent victimization (OR=5.81, 95%CI=3.45, 9.78) and a history of parental violent crime (OR=1.37, 95%CI=1.15, 1.63; Table 2). Overall, the premorbid domain was associated with an increased risk of violence (OR=1.79, 95%CI=1.16, 2.77; Figure 1). In addition, parental bereavement, ever being non-violently victimized, childhood abuse, and a parental history of substance misuse were associated with violence risk, but reported in only two independent samples (Table S4).

### Psychopathological domain

A diagnosis of schizophrenia was associated with an increased risk of violence compared with other psychotic disorders in a given study (OR=1.63, 95%CI=1.17, 2.28; Table 2), as was comorbid diagnosis of personality disorder (OR=2.30, 95%CI=1.71, 3.09) and number of past hospitalizations (OR=2.65, 95%CI=1.45, 4.84). There was also an association of the overall psychopathological domain (OR=1.66, 95%CI=1.05, 2.62; Figure 1). A lack of insight, comorbid diagnosis of antisocial personality disorder, a diagnosis of bipolar disorder, younger age of psychosis onset, and traumatic brain injury were associated with violence in two independent samples (Table S4).

### Sociodemographic domain

No educational qualifications versus any (OR=1.46, 95%CI=1.26, 1.69), Black and minority ethnicity (OR=1.72, 95%CI=1.08, 2.74), and male gender (OR=1.67, 95%CI=1.06, 2.64) were associated with higher violence risk (Table 2), as was the overall sociodemographic domain (OR=1.61, 95%CI=1.07, 2.44).

### Substance misuse domain

All factors in the substance misuse domain were associated with an increased risk of violence. Substance misuse, drug misuse (current or recent), alcohol misuse, and a history of alcohol misuse had similar effect sizes (ORs ranging from 1.61 to 2.41). The overall substance misuse domain was associated with an increased risk of violence (OR=2.36, 95%CI=1.99, 2.80; Figure 1). Cannabis use history and recent alcohol misuse were reported in two samples, with the highest OR estimated for a history of cannabis use (OR=3.34, 95%CI=2.32, 4.82; Table S4).

### Suicidality domain

Self-harm history was associated with an elevated violence risk (OR=1.74, 95%CI=1.01, 2.98; Table 2; Figure 1). Unintentional self-harm, reported in two samples, was also associated with violence (OR=5.50, 95%CI=4.26, 7.08; Table S4).

### Treatment related domain

Antipsychotic medication was associated with a reduced risk of violence (OR=0.51, 95%CI=0.27, 0.96; Table 2). Treatment adherence had a protective association, though not statistically significant. Antidepressant treatment was also associated with a protective effect (OR=0.80, 95%CI=0.66, 0.97; Table S4) though it was only reported in two samples (Table S4).

### Sensitivity analyses

When restricting analyses to those samples where a majority of patients were from inpatient settings (k=10 or 24% of the overall sample), risk factors occurring in two or more of these samples all had non-significant associations apart from the number of previous hospitalizations, reported in two independent samples (OR=2.07, 95%CI=1.22, 3.53; Table 3). Overall, point estimates in inpatient settings were also reduced as compared to the main analysis. Meanwhile, only including samples from community settings did not materially change results (Table S5). When restricting analyses to samples where violence was defined by conviction or arrest for violence/homicide (k=14), there were 11 factors reported in k≥3 independent samples, and findings were similar to the main analyses (Table S6). There were no material changes when excluding risk factors where associations were reported as HRs or probit regression coefficients (Table S7). Results were also similar when restricting analyses to studies with independent samples that had quality scores ≥75% (Table S8). We found no evidence of publication bias.

### Meta-regression

For the majority of included risk factors, heterogeneity as measured by the I^2^ statistic was high. Meta-regression was conducted for risk factors that occurred in at least seven independent samples and that had an I^2^ ≥75%. These risk factors include: unemployment, self-harm history, alcohol misuse, male gender, substance misuse, drug misuse, and violence history. We only found statistically significant results for male gender. In univariate meta-regression, we found that if the study reported the outcome as violent arrest or violent conviction, then being male was statistically significantly associated with a greater risk of violence compared to when the study used a less severe definition of violence. This was also the case for samples based in Europe compared with other regions. In samples where there was a higher percentage of men, being male was associated with a lower violence risk as compared to samples where there was a lower percentage of men. None of these sample characteristics retained statistical significance when simultaneously entered into a multivariable meta-regression model.

## Discussion

In this updated systematic review and meta-analysis of risk factors for violence in psychosis based on longitudinal studies, we identified 41 independent samples comprising 203,297 individuals. We examined 30 individual risk factors that were reported in at least three independent samples. This synthesis identified novel risk factors and validates associations identified in previous work, providing information on several potentially modifiable risk factors. Importantly, the focus on longitudinal studies reduces the likelihood of reverse causation, meaning that findings can inform risk stratification and help identify potential treatment targets.

We found that criminal history was the risk factor domain with the strongest association with violence, followed by the substance misuse domain. In relation to criminal history, both previous violent and non-violent crime were risk factors – possibly because these measure a general propensity for criminality, or because engaging in criminality introduces individuals to social contexts and networks that increase the risk of subsequent violence. There are many explanations for the importance of comorbid substance misuse as a risk factor domain. Intoxication leads to poorer impulse control, which has been found to be a strong risk factor for violence in cross-sectional studies.^11^ Individuals with schizophrenia (and other severe mental illnesses, including bipolar disorder) may also self-medicate with substances to manage symptoms,^66^ possibly affecting treatment adherence and effectiveness,^67^ while being an indicator of more severe symptomatology. Drug misuse may additionally be an entry route into criminality and expose individuals to violent environments.^68 69^ Another important risk factor domain is suicidality. It is likely that common processes underpin violence and suicidality – for example, they may both be outward expressions to regulate intense internal states.^68^ The strength of the association for these and other studied risk factor domains were broadly similar to the previous review,^11^ validating the evidence using longitudinal designs.

An unexpected finding in this review was the lack of a clear association between overall positive symptom scores and violence, despite previous research finding that positive symptoms are an important risk factor for violence.^70^ However, the confidence interval for our result was wide (OR=1.12, 95% CI=0.45, 2.82), and with a high I^2^ statistic. We also found a modest, though statistically non-significant, association for negative symptom score (OR=1.10, 95%CI=0.95, 1.27). While this may be consistent with the null association found in the previous review (OR=1.00, 95%CI=0.90, 1.20),^11^ another possibility is that negative symptoms are a marker of disease severity and partial response to treatment. It is also possible that other symptoms associated with violence, such as hostility, are misclassified as negative symptoms due to their overlap with blunted affect and asociality.^71^

Antipsychotic treatment was statistically significantly associated with reduced violence (OR=0.51, 95%CI=0.27, 0.96), in line with the protective effect expected from interventions that reduce psychotic symptoms. We also found that treatment with an antidepressant (reported in two independent samples) was a protective factor (0.80, 95%CI=0.66, 0.97). It is possible that the affective component of a psychosis is being treated with the antidepressant, including anger, which has been shown to be one mechanism for violence in schizophrenia.^70^ Alternatively, being under medical treatment may be a marker of closer contact with clinical services. Another novel finding is that cannabis use (reported in two independent samples) was identified as a risk factor for violence in the current review with an odds ratio of 3.34 (95%CI=2.32, 4.82), while it had a null association in the previous review. This suggests that cannabis use disorder could be considered as part of an individualized violence risk assessment.^72 73^ It is also possible that cannabis drives at least part of the overall association of substance misuse with violence, given that many studies did not separate out the effect of cannabis misuse from that of other substances. Previous literature has found that cannabis use is associated with earlier onset of schizophrenia and other psychotic disorders,^74–76^ and that earlier onset of psychosis is associated with greater risk of violent crime.^77^ We further identified traumatic brain injury as a potential risk factor, which was not reported in the previous review. While organic brain disorders have been implicated in violence,^78^ further research is necessary – especially as we only identified this risk factor in two independent samples.

### Clinical implications

Our results confirm the importance of previous crime and previous or current substance misuse, and also identify potential novel risk factors including cannabis misuse. These findings can inform more precise stratification of violence risk for patients with psychotic disorders. However, the clinical impact of accurate risk prediction models depends on whether effective interventions are available. Many of the identified risk factors, such as comorbid substance misuse, are potential targets for clinical intervention if found to be causal. Others may be markers of modifiable risk factors. For example, criminal history could be a marker of pro-criminal beliefs that may be reduced using cognitive or behavioural interventions.^68^ Further research is recommended to test the impact of different types of interventions in individuals with psychosis.

We have included multiple study settings, and it is possible that certain risk factors vary in their association depending on the context in which the assessment is made. When we restricted our analysis to inpatient settings, risk factors were attenuated and became statistically non-significant. Patients from inpatient settings are likely to be a selected sample due to greater severity of symptoms and may have more similar risk factor profiles, leading to lower variance in violence risk. Consequently, more in-depth risk assessment may be necessary.

### Strengths and limitations

We have focused on longitudinal studies, which reduces the possibility that reverse causation explains the results. However, several limitations should be noted. First, we relied on minimally adjusted effect estimates, given that covariate adjustment varied across studies. This may help explain why the effect estimates are quite similar within certain domains (notably the criminal history domain). Different types of criminality often co-occur within individuals – the presence of a given type may therefore be a marker of another, obscuring the relationship of specific risk factors with violence. We did not have individual participant data, which may have allowed us to provide consistently adjusted effect estimates.^79^ Second, causal inferences are not possible, given that we extract minimally adjusted effect estimates and include a mix of study designs with different aims (descriptive, causal, and predictive). Nonetheless, our results may offer insight into risk factors that are important to consider in predictive and causal models, and – if found to be causal – that are modifiable in the sense that they can be improved through clinical intervention. Both types of risk factors are important – causal factors would allow for interventions to mitigate risk, while predictive ones would provide better risk stratification.

Third, our decision to pool individual risk factors and risk factor domains need to be considered in the context of substantial heterogeneity, e.g. in terms of study designs and measures. However, pooling can provide a broad overview of a large body of evidence.^11^ Fourth, some risk factors were only measured in a cross-sectional setting, notably those based on detailed neuropsychological or cognitive tests or biomarkers, which meant that they were not examined. Fifth, we did not use a second reviewer for screening, given the large number of references at the screening phase and because we followed a previous protocol. However, the original search and the updated one were carried out by independent reviewers. Sixth, included studies predominantly come from high-income Western countries and further research is needed to better understand risk factors in other regions.

### Conclusion

In this updated systematic review and meta-analysis using longitudinal designs, we have validated the importance of comorbid substance misuse and criminal history as risk factors for violence in psychosis, while identifying potential novel risk factors such as cannabis misuse that require replication. If found to be causal, several of these factors represent modifiable intervention targets.

## Supplementary Material

Supplementary material

## Figures and Tables

**Table 1:** Characteristics of independent samples^a^

	Original search	Updated search	Overall
	(N=21)	(N=20)	(N=41)
Publication Year			
Median [Min, Max]	2004 [1983, 2010]	2016 [2013, 2022]	2011 [1983, 2022]
Region			
Asia	1 (4.8%)	0 (0%)	1 (2.4%)
Australia	0 (0%)	1 (5.0%)	1 (2.4%)
Europe	8 (38.1%)	12 (60.0%)	20 (48.8%)
International collaboration	3 (14.3%)	1 (5.0%)	4 (9.8%)
UK	2 (9.5%)	3 (15.0%)	5 (12.2%)
USA	7 (33.3%)	3 (15.0%)	10 (24.4%)
Sample size			
Median [Min, Max]	207 [16, 4,035]	1221.5 [30, 58,771]	404 [16, 58,771]
Inpatient population			
No	12 (57.1%)	19 (95.0%)	31 (75.6%)
Yes	9 (42.9%)	1 (5.0%)	10 (24.4%)
Violence measure			
Aggression	1 (4.8%)	0 (0%)	1 (2.4%)
Attack	10 (47.6%)	2 (10.0%)	12 (29.3%)
Attack / verbal abuse	4 (19.0%)	6 (30.0%)	10 (24.4%)
Forensic care	0 (0%)	1 (5.0%)	1 (2.4%)
Homicide conviction	0 (0%)	1 (5.0%)	1 (2.4%)
PANSS^b^ hostility (continuous)	0 (0%)	2 (10.0%)	2 (4.9%)
Physical assault episodes (continuous)	1 (4.8%)	0 (0%)	1 (2.4%)
Violent offence arrest	0 (0%)	1 (5.0%)	1 (2.4%)
Violent offence conviction	5 (23.8%)	7 (35.0%)	12 (29.3%)

a For overlapping samples, information from the sample with the greatest sample size is presented

b PANSS: The Positive and Negative Syndrome Scale

**Table 2:** Pooled odds ratios of risk factors for violence in psychosis

Risk factor domain	Risk factor^a,b,c^	K	No. Violent	No. Total	OR (95% CI)	Z-value	I^2^
Criminal history	Violence history	15	5,521	111,504	2.91 (2.06, 4.10)	6.07	96
	Criminal history: prison	3	1,756	10,140	3.04 (2.08, 4.44)	5.76	60
	Violence history: recent	3	1,564	9,272	3.40 (1.65, 7.00)	3.32	78
	Non-violent crime: history	6	2,478	30,154	4.16 (1.66, 10.38)	3.05	99
Negative symptoms	Negative: PANSS (continuous)	4	192	1,767	1.10 (0.95, 1.27)	1.23	0
Positive symptoms	Positive symptom score: PANSS (continuous)	5	192	2,921	1.12 (0.45, 2.82)	0.25	99
Premorbid	Victimization: violent, recent	5	212	67,623	5.81 (3.45, 9.78)	6.62	67
	Family history: violent crime, parent	4	4,525	97,260	1.37 (1.15, 1.63)	3.47	47
Psychopathological	Diagnosis: personality disorder	4	461	3,246	2.30 (1.71, 3.09)	5.49	23
	Hospitalization: history, number (continuous)	3	128	386	2.65 (1.45, 4.84)	3.18	27
	Diagnosis: schizophrenia	4	70	910	1.63 (1.17, 2.28)	2.87	0
	Total: PANSS (continuous)	3	181	1,655	0.67 (0.15, 2.95)	-0.53	97
Sociodemographic	Education: no qualifications vs any	3	940	7,697	1.46 (1.26, 1.69)	5.00	4
	Black and minority ethnicity	4	259	2,430	1.72 (1.08, 2.74)	2.28	68
	Gender: male	12	2,953	89,379	1.67 (1.06, 2.64)	2.19	96
	Age: younger	4	983	59,827	1.63 (0.80, 3.32)	1.35	91
	SES: low income	6	4,110	49,864	1.11 (0.84, 1.47)	0.76	93
	Living situation: homeless	3	293	1,393	1.31 (0.59, 2.92)	0.67	0
	Marital status: single	11	2,917	31,384	1.07 (0.84, 1.35)	0.53	72
	Living situation: living with others	3	202	1,883	1.10 (0.72, 1.68)	0.45	0
	Employment: unemployed	7	377	3,353	0.86 (0.39, 1.91)	-0.36	90
	Living situation: living alone	3	322	2,378	0.75 (0.57, 1.00)	-1.97	0
Substance misuse	Substance misuse	11	2,929	35,209	2.41 (1.84, 3.15)	6.42	87
	Drug misuse	11	3,935	122,048	2.20 (1.72, 2.82)	6.28	86
	Alcohol misuse: history	3	1,009	59,873	1.61 (1.31, 1.99)	4.45	19
	Drug misuse: recent	4	254	2,320	1.80 (1.35, 2.38)	4.06	0
	Alcohol misuse	7	3,630	34,453	1.92 (1.38, 2.68)	3.86	87
Suicidality	Self-harm: history	9	3,324	155,356	1.74 (1.01, 2.98)	1.99	97
Treatment related	Medication: treatment adherence	4	261	2,575	0.59 (0.33, 1.06)	-1.77	80
	Medication: antipsychotic	4	2,272	70,459	0.51 (0.27, 0.96)	-2.09	95

a Recent: within last year

b PANSS: The Positive and Negative Syndrome Scale

c SES: Socioeconomic status

**Table 3:** Pooled odds ratios of risk factors for violence in psychosis in inpatient settings

Risk factor domain	Risk factor^a,b^	K	No. Violent	No. Total	OR (95% CI)	Z-value	I^2^
Criminal history	Violence history	3	215	586	1.23 (0.35, 4.33)	0.33	83
Negative symptoms	Negative: PANSS (continuous)	2	104	220	1.42 (0.62, 3.23)	0.83	64
Positive symptoms	Paranoia: BPRS (continuous)	2	7	223	3.07 (0.88, 10.69)	1.76	52
	Positive symptom score: PANSS (continuous)	2	104	220	0.48 (0.07, 3.29)	-0.75	92
Psychopathological	Hospitalization: history, number (continuous)	2	106	201	2.07 (1.22, 3.53)	2.69	0
	Total: PANSS (continuous)	2	104	220	0.41 (0.05, 3.64)	-0.80	94
Sociodemographic	Marital status: single	3	131	1,922	1.22 (0.84, 1.77)	1.03	0
	Gender: male	3	267	3,105	1.59 (0.44, 5.81)	0.70	92
	Living situation: living with others	2	125	448	1.28 (0.46, 3.55)	0.47	0
	Living situation: homeless	2	125	448	0.54 (0.08, 3.88)	-0.61	0
	Employment: unemployed	2	69	367	0.33 (0.01, 10.01)	-0.64	94
Suicidality	Self-harm: history	2	69	367	1.64 (0.88, 3.05)	1.57	0

a PANSS: The Positive and Negative Syndrome Scale

b BPRS: Brief Psychiatric Rating Scale

## Data Availability

Data availability is not applicable to this article as no new data were created or analysed in this study.

## Analytic Code Availability

The analytic code for running the meta-analysis is available upon request.

## References

[1] Dolan P, Loomes G, Peasgood T, Tsuchiya A (2005). Estimating the intangible victim costs of violent crime. British Journal of Criminology.

[2] McCollister KE, French MT, Fang H (2010). The cost of crime to society: New crime-specific estimates for policy and program evaluation. Drug and alcohol dependence.

[3] Barbui C, Saraceno B (2015). Closing forensic psychiatric hospitals in Italy: a new revolution begins?. British Journal of Psychiatry.

[4] Whiting D, Gulati G, Geddes JR, Fazel S (2022). Association of schizophrenia spectrum disorders and violence perpetration in adults and adolescents from 15 countries: a systematic review and meta-analysis. JAMA Psychiatry.

[5] Fazel S, Wolf A, Palm C, Lichtenstein P (2014). Violent crime, suicide, and premature mortality in patients with schizophrenia and related disorders: A 38-year total population study in Sweden. Lancet Psychiatry.

[6] Whiting D, Gulati G, Geddes JR, Dean K, Fazel S (2024). Violence in schizophrenia: triangulating the evidence on perpetration risk. World psychiatry.

[7] Whiting D, Lennox BR, Fazel S (2020). Violent outcomes in first-episode psychosis: A clinical cohort study. Early Intervention in Psychiatry.

[8] Troquete NAC, Van Den Brink RHS, Beintema H, Mulder T, Van Os TWDP, Schoevers RA (2013). Risk assessment and shared care planning in out-patient forensic psychiatry: Cluster randomised controlled trial. British Journal of Psychiatry.

[9] Ramesh T, Igoumenou A, Vazquez Montes M, Fazel S (2018). Use of risk assessment instruments to predict violence in forensic psychiatric hospitals: a systematic review and meta-analysis. European Psychiatry.

[10] Tyrer P, Sharfstein S, O’Reilly R, Allison S, Bastiampillai T (2017). Psychiatric hospital beds: an Orwellian crisis. The Lancet.

[11] Witt K, Van Dorn R, Fazel S (2013). Risk factors for violence in psychosis: systematic review and meta-regression analysis of 110 studies. PloS one.

[12] Broderick C, Azizian A, Kornbluh R, Warburton K (2015). Prevalence of physical violence in a forensic psychiatric hospital system during 2011-2013: Patient assaults, staff assaults, and repeatedly violent patients. CNS spectrums.

[13] Shmueli G (2010). To Explain or to Predict?. Statistical Science.

[14] Chakrabarti S, Singh N (2022). Psychotic symptoms in bipolar disorder and their impact on the illness: A systematic review. World J Psychiatry.

[15] Favril L, Yu R, Hawton K, Fazel S (2020). Risk factors for self-harm in prison: a systematic review and meta-analysis. Lancet Psychiatry.

[16] VanderWeele TJ (2020). Optimal approximate conversions of odds ratios and hazard ratios to risk ratios. Biometrics.

[17] Luchini C, Stubbs B, Solmi M, Veronese N (2017). Assessing the quality of studies in meta-analyses: Advantages and limitations of the Newcastle Ottawa Scale. World Journal of Meta-Analysis.

[18] Zapf A, Castell S, Morawietz L, Karch A (2016). Measuring inter-rater reliability for nominal data–which coefficients and confidence intervals are appropriate?. BMC medical research methodology.

[19] Peters JL, Sutton AJ, Jones DR, Abrams KR, Rushton L (2006). Comparison of Two Methods to Detect Publication Bias in Meta-analysis. JAMA.

[20] Arango C, Barba AC, González-Salvador T, Ordonez AC (1999). Violence in inpatients with schizophrenia: a prospective study. Schizophrenia bulletin.

[21] Baird A, Shaw J, Hunt IM, Kapur N, Appleby L, Webb RT (2018). National study comparing the characteristics of patients diagnosed with schizophrenia who committed homicide vs. those who died by suicide. Journal of Forensic Psychiatry & Psychology.

[22] Baird A, Webb RT, Hunt IM, Appleby L, Shaw J (2020). Homicide by men diagnosed with schizophrenia: National case-control study. BJPsych Open.

[23] Beaudoin M, Potvin S, Giguère CE, Discepola SL, Dumais A (2020). Persistent cannabis use as an independent risk factor for violent behaviors in patients with schizophrenia. NPJ Schizophrenia.

[24] Bitter I, Czobor P, Dossenbach M, Volavka J (2005). Effectiveness of clozapine, olanzapine, quetiapine, risperidone, and haloperidol monotherapy in reducing hostile and aggressive behavior in outpatients treated for schizophrenia: a prospective naturalistic study (IC-SOHO). European Psychiatry.

[25] Buchanan A, Sint K, Swanson J, Rosenheck R (2019). Correlates of future violence in people being treated for schizophrenia. American Journal of Psychiatry.

[26] Bulgari V, Iozzino L, Ferrari C, Picchioni M, Candini V, De Francesco A (2017). Clinical and neuropsychological features of violence in schizophrenia: A prospective cohort study. Schizophrenia research.

[27] Cannon M, Huttunen MO, Anen AJT, Arseneault L, Jones PB, Murray RM (2002). Perinatal and childhood risk factors for later criminality and violence in schizophrenia: longitudinal, population-based study. The British Journal of Psychiatry.

[28] Coid JW, Ullrich S, Kallis C, Freestone M, Gonzalez R, Bui L (2016). Improving risk management for violence in mental health services: a multimethods approach.

[29] Cuffel BJ, Shumway M, Chouljian TL, Macdonald T (1994). A longitudinal study of substance use and community violence in schizophrenia. The Journal of Nervous and Mental Disease.

[30] Dean K, Walsh E, Moran P, Tyrer P, Creed F, Byford S (2006). Violence in women with psychosis in the community: prospective study. The British Journal of Psychiatry.

[31] Eriksson Å, Romelsjö A, Stenbacka M, Tengström A (2011). Early risk factors for criminal offending in schizophrenia: a 35-year longitudinal cohort study. Social psychiatry and psychiatric epidemiology.

[32] Faay MDM, van Os J (2020). Aggressive Behavior, Hostility, and Associated Care Needs in Patients With Psychotic Disorders: A 6-Year Follow-Up Study. Frontiers in Psychiatry.

[33] Fazel S, Gratin M, Carlstrom E, Lichtenstein P, Langstrom N (2009). Risk factors for violent crime in Schizophrenia: a national cohort study of 13,806 patients. Journal of clinical psychiatry.

[34] Fazel S, Buxrud P, Ruchkin V, Grann M (2010). Homicide in discharged patients with schizophrenia and other psychoses: a national case-control study. Schizophrenia research.

[35] Fazel S, Wolf A, Larsson H, Lichtenstein P, Mallett S, Fanshawe TR (2017). Identification of low risk of violent crime in severe mental illness with a clinical prediction tool (Oxford Mental Illness and Violence tool [OxMIV]): a derivation and validation study. Lancet Psychiatry.

[36] Foley SR, Kelly BD, Clarke M, McTigue O, Gervin M, Kamali M (2005). Incidence and clinical correlates of aggression and violence at presentation in patients with first episode psychosis. Schizophrenia research.

[37] Hachtel H, Harries C, Luebbers S, Ogloff JRP (2018). Violent offending in schizophrenia spectrum disorders preceding and following diagnosis. Australian & New Zealand Journal of Psychiatry.

[38] Haddock G, Eisner E, Davies G, Coupe N, Barrowclough C (2013). Psychotic symptoms, self-harm and violence in individuals with schizophrenia and substance misuse problems. Schizophrenia research.

[39] Herrera JN, Sramek JJ, Costa JF, Roy S, Heh CW, Nguyen BN (1988). High potency neuroleptics and violence in schizophrenics. The Journal of nervous and mental disease.

[40] Hodgins S, Hiscoke UL, Freese R (2003). The antecedents of aggressive behavior among men with schizophrenia: a prospective investigation of patients in community treatment. Behavioral Sciences & the Law.

[41] Krakowski M, Czobor P (2004). Gender differences in violent behaviors: relationship to clinical symptoms and psychosocial factors. American Journal of Psychiatry.

[42] Langeveld J, Bjørkly S, Auestad B, Barder H, Evensen J, Ten Velden Hegelstad W (2014). Treatment and violent behavior in persons with first episode psychosis during a 10-year prospective follow-up study. Schizophrenia research.

[43] Lincoln TM, Hodgins S (2008). Is lack of insight associated with physically aggressive behavior among people with schizophrenia living in the community?. The Journal of nervous and mental disease.

[44] Munkner R, Haastrup S, Joergensen T, Kramp P (2005). Incipient offending among schizophrenia patients after first contact to the psychiatric hospital system. European Psychiatry.

[45] Nolan KA, Volavka J, Czobor P, Sheitman B, Lindenmayer J-P, Citrome LL (2005). Aggression and psychopathology in treatment-resistant inpatients with schizophrenia and schizoaffective disorder. Journal of psychiatric research.

[46] Pedersen CG, Olrik Wallenstein Jensen S, Johnsen SP, Nordentoft M, Mainz J (2013). Processes of in-hospital psychiatric care and subsequent criminal behaviour among patients with schizophrenia: a national population-based, follow-up study. Canadian Journal of Psychiatry.

[47] Piontek K, Kutscher SU, König A, Leygraf N (2013). History of treatment of schizophrenic forensic patients prior to admission: a comparison with schizophrenic general psychiatric patients. Der Nervenarzt.

[48] Räsänen P TJ, Isohanni M, Rantakallio P, Lehtonen J (1998). Schizophrenia, alcohol abuse, violent behavior: A 26-year followup study of an unselected birth cohort. Schizophrenia Bulletin.

[49] Rolin SA, Scodes J, Dambreville R, Nossel IR, Bello I, Wall MM (2022). Feasibility and Utility of Different Approaches to Violence Risk Assessment for Young Adults Receiving Treatment for Early Psychosis. Community mental health journal.

[50] Sariaslan A, Lichtenstein P, Larsson H, Fazel S (2016). Triggers for Violent Criminality in Patients With Psychotic Disorders. JAMA Psychiatry.

[51] Singh JP, Grann M, Lichtenstein P, Långström N, Fazel S (2012). A novel approach to determining violence risk in schizophrenia: Developing a stepped strategy in 13,806 discharged patients. PLoS ONE.

[52] Soyka M, Graz C, Bottlender R, Dirschedl P, Schoech H (2007). Clinical correlates of later violence and criminal offences in schizophrenia. Schizophrenia research.

[53] Steinert T, Wiebe C, Gebhardt RP (1999). Aggressive behavior against self and others among first-admission patients with schizophrenia. Psychiatric Services.

[54] Swanson JW, Swartz MS, Wagner HR, Burns BJ, Borum R, Hiday VA (2000). Involuntary out-patient commitment and reduction of violent behaviour in persons with severe mental illness. The British Journal of Psychiatry.

[55] Swanson JW, Swartz MS, Elbogen EB (2004). Effectiveness of atypical antipsychotic medications in reducing violent behavior among persons with schizophrenia in community-based treatment. Schizophrenia bulletin.

[56] Thomas S, Leese M, Walsh E, McCrone P, Moran P, Burns T (2005). A comparison of statistical models in predicting violence in psychotic illness. Comprehensive psychiatry.

[57] Webb RT, Lichtenstein P, Larsson H, Geddes JR, Fazel S (2014). Suicide, hospital-presenting suicide attempts, and criminality in bipolar disorder: Examination of risk for multiple adverse outcomes. Journal of Clinical Psychiatry.

[58] Witt K, Hawton K, Fazel S (2014). The relationship between suicide and violence in schizophrenia: analysis of the Clinical Antipsychotic Trials of Intervention Effectiveness (CATIE) dataset. Schizophrenia research.

[59] Witt K, Lichtenstein P, Fazel S (2015). Improving risk assessment in schizophrenia: epidemiological investigation of criminal history factors. British Journal of Psychiatry.

[60] Wootton L, Buchanan A, Leese M, Tyrer P, Burns T, Creed F (2008). Violence in psychosis: Estimating the predictive validity of readily accessible clinical information in a community sample. Schizophrenia research.

[61] Yen C-F, Yeh M-L, Chen C-S, Chung H-H (2002). Predictive value of insight for suicide, violence, hospitalization, and social adjustment for outpatients with schizophrenia: a prospective study. Comprehensive Psychiatry.

[62] Yesavage JA (1983). Inpatient violence and the schizophrenic patient: A study of Brief Psychiatric Rating Scale scores and inpatient behavior. Acta Psychiatrica Scandinavica.

[63] Yesavage JA (1984). Correlates of dangerous behavior by schizophrenics in hospital. Journal of Psychiatric Research.

[64] Volavka J, Van Dorn RA, Citrome L, Kahn RS, Fleischhacker WW, Czobor P (2016). Hostility in schizophrenia: An integrated analysis of the combined Clinical Antipsychotic Trials of Intervention Effectiveness (CATIE) and the European First Episode Schizophrenia Trial (EUFEST) studies. European Psychiatry.

[65] Oluwoye O, Monroe-DeVita M, Burduli E, Chwastiak L, McPherson S, McClellan JM (2019). Impact of tobacco, alcohol and cannabis use on treatment outcomes among patients experiencing first episode psychosis: Data from the national RAISE-ETP study. Early intervention in psychiatry.

[66] Gregg L, Barrowclough C, Haddock G (2007). Reasons for increased substance use in psychosis. Clinical psychology review.

[67] Lindsey WT, Stewart D, Childress D (2012). Drug interactions between common illicit drugs and prescription therapies. The American journal of drug and alcohol abuse.

[68] Lambe S, Cooper K, Fazel S, Freeman D (2023). Psychological framework to understand interpersonal violence by forensic patients with psychosis. The British Journal of Psychiatry.

[69] Zhong S, Yu R, Fazel S (2020). Drug Use Disorders and Violence: Associations with Individual Drug Categories. Epidemiologic Reviews.

[70] Coid JW, Kallis C, Doyle M, Shaw J, Ullrich S (2018). Shifts in positive and negative psychotic symptoms and anger: effects on violence. Psychological medicine.

[71] Correll CU, Schooler NR (2020). Negative Symptoms in Schizophrenia: A Review and Clinical Guide for Recognition, Assessment, and Treatment. Neuropsychiatr Disease and Treatment.

[72] Dellazizzo L, Potvin S, Athanassiou M, Dumais A (2020). Violence and Cannabis Use: A Focused Review of a Forgotten Aspect in the Era of Liberalizing Cannabis. Frontiers in Psychiatry.

[73] Dellazizzo L, Potvin S, Beaudoin M, Luigi M, Dou BY, Giguère C (2019). Cannabis use and violence in patients with severe mental illnesses: A meta-analytical investigation. Psychiatry Research.

[74] Hjorthøj C, Compton W, Starzer M, Nordholm D, Einstein E, Erlangsen A (2023). Association between cannabis use disorder and schizophrenia stronger in young males than in females. Psychological Medicine.

[75] Large M, Sharma S, Compton MT, Slade T, Nielssen O (2011). Cannabis use and earlier onset of psychosis: a systematic meta-analysis. Archives of general psychiatry.

[76] Di Forti M, Sallis H, Allegri F, Trotta A, Ferraro L, Stilo SA (2014). Daily use, especially of high-potency cannabis, drives the earlier onset of psychosis in cannabis users. Schizophrenia bulletin.

[77] Moulin V, Alameda L, Framorando D, Baumann P-S, Gholam M, Gasser J (2020). Early onset of cannabis use and violent behavior in psychosis. European psychiatry.

[78] Fazel S, Philipson J, Gardiner L, Merritt R, Grann M (2009). Neurological disorders and violence: a systematic review and meta-analysis with a focus on epilepsy and traumatic brain injury. Journal of neurology.

[79] Riley RD, Lambert PC, Abo-Zaid G (2010). Meta-analysis of individual participant data: rationale, conduct, and reporting. Bmj.

