# Supplementary material for: Systematic review of risk factors for violence in psychosis: a 10-year update
Source: Br J Psychiatry. Author manuscript; Available in PMC 2025 Mar 19. (PMC7617503; doi:10.1192/bjp.2024.120)
Supplement: Supplementary material [file EMS198181-supplement-Supplementary_material.pdf]

Supplement: Risk factors for violence in psychosis

Table of Contents

Figure S1. Flowchart of study inclusion 2

Table S1. Search term 3

Table S2. Reasons for study exclusion 4

Table S3. Characteristics of included studies 5

Table S4. Pooled odds ratios of risk factors that occur in only two independent samples 10

Table S5. Pooled odds ratios of risk factors for violence in psychosis outside of inpatient settings..12

Table S6. Pooled odds ratios of risk factors for homicide/violent offence conviction or arrest in psychosis 13

Table S7. Pooled odds ratios of risk factors for violence in psychosis when effect estimates reported as hazard ratios or probit regression coefficients are excluded 14

Table S8. Pooled odds ratios of risk factors for violence in psychosis when including only high quality studies 16

Figure S1. Flowchart of study inclusion

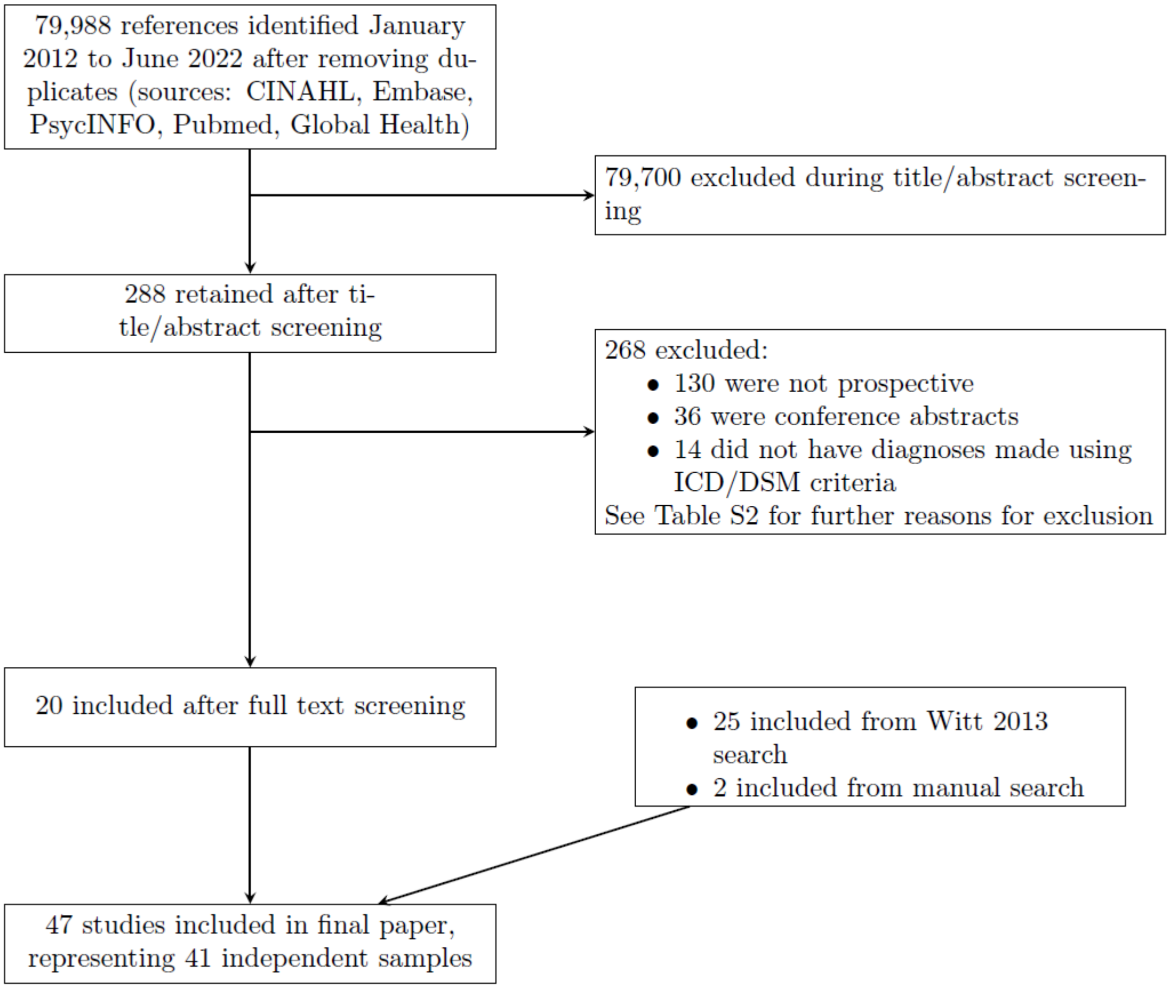

Table S1. Search term

Operator	Term	Where?
	(schiz* AND (viol* OR aggress* OR crim* OR offend* OR danger* OR hosti*))	Title/Abstract
OR	(psych* AND (viol* OR aggress* OR crim* OR offend* OR danger* OR hosti*))	Title/Abstract
OR	(mental* AND (viol* OR aggress* OR crim* OR offend* OR danger* OR hosti*))	Title/Abstract

Table S2. Reasons for study exclusion

Exclusion reason	Number of studies
Design is not longitudinal	130
Conference abstract only	36
Psychosis diagnosis not determined by ICD or DSM criteria[a]	14
Study population consists solely of individuals with violence histories	12
No informative risk factors	11
No separation of violent from non-violent offending	10
A majority of study population not diagnosed with psychosis	8
No violence outcome	8
No individual risk factors	6
No reporting/analysis within psychosis group	5
Review	5
Self-harm/suicide is included in the outcome	5
Compares types of violence to each other only	4
Commentary	3
Letter to editor	2
No access	2
Addendum	1
Case series	1
Editorial only	1
Protocol	1
Thesis write-up of studies already included	1
Only transient psychosis included in study population	1
Only violence outcome is violent ideation	1

[a] ICD: International Classification of Diseases ; DSM: Diagnostic and Statistical Manual of Mental Disorders

Table S3. Characteristics of included studies

First author	Year	Region	No. Total	No. Violent	Diagnosis	Diagnosis criteria	Violence measure	Scale of violence measure	Majority (>95%) current inpatients?	Quality (%)
Arango	1999	Europe	63	16	Schizophrenia, schizoaffective disorder	DSM-IV	Attack	Overt Aggression Scale	Yes	53%
Baird	2018	UK	945	168	Schizophrenia, schizophreniform disorder, schizoaffective disorder, delusional disorder or psychosis otherwise not specified	DSM-IV, ICD-10	Homicide conviction		No	54%
Baird	2020	UK	702	160	Schizophrenia, schizotypal, and delusional disorders	ICD-10	Homicide conviction		No	67%
Beaudoin	2020	USA	965	85	Schizophrenia	DSM-IV	Attack / verbal abuse	Macarthur Community Violence	No	71%
Bitter	2005	International collaboration	3,135	362	Schizophrenia	ICD-10, DSM-IV	Aggression		No	67%
Buchanan	2019	USA	1,435	77	Schizophrenia	DSM-IV	Attack	Macarthur Community Violence	No	73%
Bulgari	2017	Europe	87	28	Schizophrenia spectrum disorder	DSM-IV	Attack / verbal abuse	Modified Overt Aggression Scale	No	80%
Cannon	2002	Europe	636	36	Schizophrenia, schizoaffective disorder or schizophreniform disorder	ICD-8, ICD-9	Violent offence conviction		No	73%
Coid	2016	UK	490		Schizophrenia, Schizotypal disorder, delusional disorder, acute/transient psychosis, schizoaffective disorder, other non-affective psychoses, bipolar affective psychosis, psychotic depression, other affective psychosis	DSM-IV, ICD-10	Attack / verbal abuse	Macarthur Community Violence ; convictions / cautions	No	93%
Cuffel	1994	USA	89	13	Schizophrenia, schizoaffective disorder, schizophreniform disorder	DSM-III-R	Attack / verbal abuse		Yes	91%

First author	Year	Region	No. Total	No. Violent	Diagnosis	Diagnosis criteria	Violence measure	Scale of violence measure	Majority (>95%) current inpatients?	Quality (%)
Dean	2006	UK	304	53	Schizophrenia, schizoaffective disorder, psychosis not otherwise specified or bipolar disorder.	ICD-10	Attack		Yes	75%
Eriksson	2010	Europe	264	93	Schizophrenia	ICD-7, ICD-8, ICD-9, ICD-10	Violent offence conviction		No	75%
Faay	2020	Europe	665	66	Schizophrenia spectrum disorders and other psychotic disorders	DSM-IV-TR	Attack / verbal abuse		No	82%
Fazel	2009 MEN	Europe	8,891	1519	Schizophrenia	ICD-8, ICD-9, ICD-10	Violent offence conviction		No	92%
Fazel	2009 WOMEN	Europe	4,915	273	Schizophrenia	ICD-8, ICD-9, ICD-10	Violent offence conviction		No	92%
Fazel	2010	Europe	105	47	Schizophrenia, bipolar affective disorder, and other psychoses (including drug-induced psychoses)	ICD-9, ICD-10	Homicide conviction		No	75%
Fazel	2014 MEN	Europe	14,261	1525	Schizophrenia and other non-affective psychoses	ICD-8, ICD-9, ICD-10	Violent offence conviction		No	100%
Fazel	2014 WOMEN	Europe	9,676	261	Schizophrenia and other non-affective psychoses	ICD-8, ICD-9, ICD-11	Violent offence conviction		No	100%
Fazel	2017	Europe	58,771	830	Schizophrenia spectrum disorders, bipolar disorder	ICD-8, ICD-9, ICD-9	Violent offence conviction		No	93%
Foley	2005	Europe	157	11	Schizophrenia, delusional disorder, substance induced psychosis, psychotic depression, organic psychosis, psychosis NOS or bipolar disorder	DSM-III-R	Attack	Modified Overt Aggression Scale	No	77%
Hachtel	2018	Australia	1,289	82	Schizophrenia spectrum disorder	ICD-10	Attack		No	85%
Haddock	2013	UK	327	105	Non-affective psychotic disorder comorbid with substanc use disorder	ICD-10/DSM-IV (psychotic disorder), DSM-IV	Attack / verbal abuse	Ward Anger Rating scale	No	71%

First author	Year	Region	No. Total	No. Violent	Diagnosis	Diagnosis criteria	Violence measure	Scale of violence measure	Majority (>95%) current inpatients?	Quality (%)
						(substance abuse disorder)				
Herrera	1988	USA	16	7	Schizophrenia	DSM-III	Attack / verbal abuse	Lion's Scale of Inpatient Violence	Yes	71%
Hodgins	2003	International collaboration	112	11	Schizophrenia or schizoaffective disorder	DSM-IV	Attack	Macarthur Risk Assessment	No	53%
Krakowski	2004	USA	1,487	189	Schizophrenia, schizoaffective disorder or bipolar disorder	DSM-III-R	Attack	Modified Overt Aggression Scale	Yes	79%
Langeveld	2014	Europe	155	22	Schizophrenia, schizophreniform disorder, schizoaffective disorder, delusional disorder, mood disorder with mood incongruent psychotic features, brief psychotic disorder, or psychosis not otherwise specified	DSM-IV	Attack / verbal abuse		No	64%
Lincoln	2008	International collaboration	171	9	Schizophrenia	DSM-IV	Attack	Macarthur Community Violence	No	64%
Munkner	2005	Europe	4,035	215	Schizophrenia	ICD-8, ICD-10	Violent offence conviction		No	92%
Nolan	2005	USA	157	88	Schizophrenia, schizoaffective disorder	DSM-IV	Attack / verbal abuse	Overt Aggression Scale	Yes	38%
Oluwoye	2018	USA	404		Schizophrenia, schizoaffective disorder, schizophreniform disorder, brief psychotic disorder, or psychotic disorder not otherwise specified	DSM-IV	PANSS hostility (continuous)	PANSS hostility	No	85%
Pedersen	2013	Europe	10,757	1282	Schizophrenia	ICD-10	Violent offence arrest		No	92%
Piontek	2013	Europe	144	72	Schizophrenia spectrum disorders	ICD-10	Forensic care		Yes	69%

First author	Year	Region	No. Total	No. Violent	Diagnosis	Diagnosis criteria	Violence measure	Scale of violence measure	Majority (>95%) current inpatients?	Quality (%)
Rasanen	1998	Europe	86	11	Schizophrenia, schizophreniform disorder, schizoaffective disorder, delusional disorder, schizoid personality disorder or schizotypal personality disorder	DSM-III-R	Violent offence conviction		No	82%
Rolin	2022	USA	30	6	Schizophrenia, schizoaffective disorder, schizophreniform disorder, psychotic disorder not otherwise specified or delusional disorder	DSM-V	Attack / verbal abuse	Macarthur Community Violence ; arrests	No	69%
Sariaslan	2016 BPD	Europe	29,692		Bipolar disorder	ICD-8, ICD-9, ICD-10	Violent offence conviction		No	100%
Sariaslan	2016 SCHZ	Europe	34,903		Schizophrenia spectrum disorder	ICD-8, ICD-9, ICD-10	Violent offence conviction		No	100%
Singh	2012	Europe	6,903	887	Schizophrenia	ICD-8, ICD-9, ICD-10	Violent offence conviction		No	92%
Soyka	2007	Europe	1,555	62	Schizophrenia	ICD-9	Violent offence conviction		Yes	71%
Steinert	1999	Europe	138	90	Schizophrenia or Schizoaffective Disorder	ICD-10	Attack / verbal abuse	Modified Overt Aggression Scale	Yes	82%
Swanson	2000	USA	262	100	Schizophrenia, schizoaffective disorder, another psychosis-spectrum disorder or bipolar disorder	DSM	Attack		No	77%
Swanson	2004	USA	229		Schizophrenia, schizoaffective disorder, or schizophreniform disorderxxxv	DSM-IV	Attack	Macarthur Community Violence ; arrests	No	42%
Thomas	2005	UK	708	158	Schizophrenia, schizoaffective disorder, affective psychosis and unspecified functional psychosis	ICD-10	Attack		No	44%

First author	Year	Region	No. Total	No. Violent	Diagnosis	Diagnosis criteria	Violence measure	Scale of violence measure	Majority (>95%) current inpatients?	Quality (%)
Volavka	2016	International collaboration	1,154		Schizophrenia (CATIE, EUFEST), schizoaffective disorder, or schizophreniform disorder (EUFEST)	DSM-IV	PANSS hostility (continuous)	PANSS hostility	No	77%
Webb	2014	Europe	15,337	635	Bipolar disorder	ICD-8, ICD-9, ICD-9	Violent offence conviction		No	100%
Witt	2014 MEN	USA	1,080	97	Schizophrenia	DSM-IV	Attack / verbal abuse	Macarthur Community Violence	No	87%
Witt	2014 WOMEN	USA	380	24	Schizophrenia	DSM-IV	Attack / verbal abuse	Macarthur Community Violence	No	87%
Witt	2015 MEN	Europe	8,891	1535	Schizophrenia	ICD-8, ICD-9, ICD-9	Violent offence conviction		No	92%
Witt	2015 WOMEN	Europe	4,915	281	Schizophrenia	ICD-8, ICD-9, ICD-9	Violent offence conviction		No	92%
Wootton	2008	UK	708	158	Schizophrenia, schizoaffective disorder, affective psychosis or unspecified functional psychosis	ICD-10	Attack		No	67%
Yen	2002	Asia	73	11	Schizophrenia	DSM-IV	Attack	Violence and Suicide Assessment Scale (VASA)	No	69%
Yesavage	1983	USA	207		Schizophrenia or schizoaffective disorder	DSM-III	Physical assault episodes (continuous)	Lion's Scale of Inpatient Violence	Yes	58%
Yesavage	1984	USA	70	10	Schizophrenia or schizoaffective disorder	DSM-III	Attack	Lion's Scale of Inpatient Violence	Yes	77%

Table S4. Pooled odds ratios of risk factors that occur in only two independent samples

Risk factor domain	Risk factor[a,b,c]	K	No. Violent	No. Total	OR (95% CI)	Z-value	I^2
Criminal history	Family history: offending behaviour	2	82	1,779	3.90 (1.06, 14.38)	2.04	90
	Non-violent crime: history, recent	2	1,617	10,180	3.82 (0.87, 16.74)	1.78	96
Positive symptoms	Paranoia: BPRS (continuous)	2	7	223	3.07 (0.88, 10.69)	1.76	52
	Hostility: PANSS (continuous)	2	66	1,819	1.50 (0.84, 2.68)	1.37	85
Premorbid	Parental bereavement	2	0	64,595	4.08 (2.63, 6.33)	6.26	0
	Family history: substance misuse, parent	2	2,160	29,598	1.32 (1.15, 1.53)	3.87	0
	Childhood abuse	2	143	2,100	1.87 (1.30, 2.69)	3.38	0
	Victimization: non-violent, recent	2	159	2,724	1.50 (0.85, 2.66)	1.39	0
Psychopathological	Diagnosis: bipolar disorder	2	641	15,367	2.12 (1.77, 2.54)	8.11	0
	Diagnosis: traumatic brain injury	2	0	64,595	5.33 (3.34, 8.49)	7.03	36
	Psychosis onset: younger age (continuous)	2	231	4,098	1.12 (1.08, 1.17)	5.77	0
	Insight: lack of	2	71	1,726	2.04 (1.26, 3.30)	2.89	0
	Diagnosis: antisocial personality disorder	2	83	256	2.46 (1.23, 4.90)	2.55	0
	General psychopathology: PANSS (continuous)	2	173	1,122	1.13 (0.98, 1.32)	1.65	0
	Insight (continuous)	2	27	1,290	2.42 (0.35, 16.72)	0.90	92
	Diagnosis: OCD	2	91	995	2.55 (0.27, 24.16)	0.82	63
	Diagnosis: other psychosis	2	64	390	0.91 (0.34, 2.45)	-0.19	0
	Diagnosis: affective disorder	2	221	1,249	0.49 (0.29, 0.83)	-2.64	0
Sociodemographic	Born abroad	2	1,786	23,937	1.50 (1.38, 1.63)	9.88	0
	Immigrant	2	2,154	24,228	1.32 (1.18, 1.48)	4.84	0
	Employment: employed	2	96	1,077	1.47 (0.42, 5.17)	0.60	76
	White	2	130	1,739	0.53 (0.37, 0.76)	-3.39	0
	Sex: female	2	281	4,700	0.54 (0.39, 0.74)	-3.80	0

Risk factor domain	Risk factor[a,b,c]	K	No. Violent	No. Total	OR (95% CI)	Z-value	I^2
Substance misuse	Cannabis use: history	2	151	1,630	3.34 (2.32, 4.82)	6.48	9
	Alcohol misuse: recent	2	85	1,455	1.63 (0.63, 4.21)	1.02	81
Suicidality	Self-harm: unintentional, recent	2	0	64,595	5.50 (4.28, 7.08)	13.29	0
Treatment related	Medication: adverse effects, antipsychotic	2	47	1,259	1.23 (0.98, 1.55)	1.80	0
	Status: inpatient	2	998	59,716	0.63 (0.13, 3.01)	-0.58	97
	Medication: antidepressant	2	990	59,473	0.80 (0.66, 0.97)	-2.23	0

[a] Recent: within last year

[b] BPRS: Brief Psychiatric Rating Scale

[c] PANSS: The Positive and Negative Syndrome Scale

Table S5. Pooled odds ratios of risk factors for violence in psychosis outside of inpatient settings

Risk factor domain	Risk factor[a,b]	K	No. Violent	No. Total	OR (95% CI)	Z-value	I^2
Criminal history	Violence history	12	5,306	110,918	3.44 (2.53, 4.66)	7.96	94
	Non-violent crime: history	6	2,478	30,154	4.16 (1.66, 10.38)	3.05	99
Positive symptoms	Positive symptom score: PANSS (continuous)	3	88	2,701	1.72 (0.81, 3.62)	1.42	90
Premorbid	Victimization: violent, recent	4	159	67,319	6.91 (4.16, 11.48)	7.47	58
	Family history: violent crime, parent	4	4,525	97,260	1.37 (1.15, 1.63)	3.47	47
Psychopathological	Diagnosis: personality disorder	3	408	2,942	2.11 (1.61, 2.76)	5.43	0
	Diagnosis: schizophrenia	3	17	606	1.58 (1.06, 2.35)	2.23	0
Sociodemographic	Non-white	4	259	2,430	1.72 (1.08, 2.74)	2.28	68
	Sex: male	9	2,686	86,274	1.72 (1.06, 2.77)	2.22	95
	Age: younger	3	930	59,523	1.46 (0.59, 3.59)	0.82	94
	SES: low income	6	4,110	49,864	1.11 (0.84, 1.47)	0.76	93
	Employment: unemployed	5	308	2,986	1.08 (0.72, 1.60)	0.36	54
	Marital status: single	8	2,786	29,462	1.05 (0.79, 1.39)	0.32	80
Substance misuse	Substance misuse	10	2,854	33,565	2.53 (1.92, 3.33)	6.59	87
	Drug misuse	10	3,882	121,744	2.19 (1.69, 2.83)	5.99	88
	Alcohol misuse: history	3	1,009	59,873	1.61 (1.31, 1.99)	4.45	19
	Drug misuse: recent	4	254	2,320	1.80 (1.35, 2.38)	4.06	0
	Alcohol misuse	6	3,577	34,149	1.98 (1.40, 2.79)	3.90	89
Suicidality	Self-harm: history	7	3,255	154,989	1.73 (0.89, 3.37)	1.61	98
Treatment related	Medication: treatment adherence	4	261	2,575	0.59 (0.33, 1.06)	-1.77	80
	Medication: antipsychotic	4	2,272	70,459	0.51 (0.27, 0.96)	-2.09	95

[a] Recent: within last year

[b] PANSS: The Positive and Negative Syndrome Scale

[c] SES: Socioeconomic status

Table S6. Pooled odds ratios of risk factors for homicide/violent offence conviction or arrest in psychosis

Risk factor domain	Risk factor[a]	K	No. Violent	No. Total	OR (95% CI)	Z-value	I^2
Criminal history	Non-violent crime: history	3	2,385	28,263	3.24 (1.99, 5.26)	4.75	96
	Violence history	7	5,026	108,025	3.10 (1.64, 5.84)	3.50	99
Premorbid	Family history: violent crime, parent	4	4,525	97,260	1.37 (1.15, 1.63)	3.47	47
Sociodemographic	Sex: male	4	2,414	82,566	3.42 (2.48, 4.70)	7.54	87
	SES: low income	5	4,033	48,429	1.29 (1.14, 1.45)	4.10	65
	Marital status: single	4	2,431	26,833	0.94 (0.65, 1.35)	-0.35	87
Substance misuse	Substance misuse	6	2,646	30,868	2.64 (1.83, 3.82)	5.17	90
	Drug misuse	7	3,764	120,512	2.21 (1.62, 3.00)	5.03	93
	Alcohol misuse	5	3,566	34,037	2.12 (1.48, 3.05)	4.06	90
Suicidality	Self-harm: history	6	3,158	153,909	1.59 (0.75, 3.37)	1.20	99
Treatment related	Medication: antipsychotic	3	2,272	70,230	0.45 (0.17, 1.18)	-1.63	98

[a] SES: Socioeconomic status

Table S7. Pooled odds ratios of risk factors for violence in psychosis when effect estimates reported as hazard ratios or probit regression coefficients are excluded

Risk factor domain	Risk factor[a,b]	K	No. Violent	No. Total	OR (95% CI)	Z-value	I^2
Criminal history	Criminal history: prison	3	1,756	10,140	3.04 (2.08, 4.44)	5.76	60
	Violence history	12	3,100	72,230	2.45 (1.68, 3.57)	4.66	90
	Violence history: recent	3	1,564	9,272	3.40 (1.65, 7.00)	3.32	78
	Non-violent crime: history	4	1,628	10,782	4.44 (1.03, 19.20)	2.00	98
Negative symptoms	Negative: PANSS (continuous)	4	192	1,767	1.10 (0.95, 1.27)	1.23	0
Positive symptoms	Positive symptom score: PANSS (continuous)	5	192	2,921	1.12 (0.45, 2.82)	0.25	99
Premorbid	Victimization: violent, recent	5	212	67,623	5.81 (3.45, 9.78)	6.62	67
Psychopathological	Diagnosis: personality disorder	4	461	3,246	2.30 (1.71, 3.09)	5.49	23
	Hospitalization: history, number (continuous)	3	128	386	2.65 (1.45, 4.84)	3.18	27
	Diagnosis: schizophrenia	4	70	910	1.63 (1.17, 2.28)	2.87	0
	Total: PANSS (continuous)	3	181	1,655	0.67 (0.15, 2.95)	-0.53	97
Sociodemographic	Non-white	4	259	2,430	1.72 (1.08, 2.74)	2.28	68
	Age: younger	4	983	59,827	1.63 (0.80, 3.32)	1.35	91
	Sex: male	10	1,431	67,139	1.39 (0.84, 2.28)	1.29	93
	Living situation: homeless	3	293	1,393	1.31 (0.59, 2.92)	0.67	0
	Living situation: living with others	3	202	1,883	1.10 (0.72, 1.68)	0.45	0
	Marital status: single	10	2,282	16,047	1.06 (0.80, 1.40)	0.39	69
	Employment: unemployed	7	377	3,353	0.86 (0.39, 1.91)	-0.36	90

Risk factor domain	Risk factor[a,b]	K	No. Violent	No. Total	OR (95% CI)	Z-value	I^2
	SES: low income	3	1,689	10,590	0.73 (0.37, 1.47)	-0.87	85
	Living situation: living alone	3	322	2,378	0.75 (0.57, 1.00)	-1.97	0
Substance misuse	Substance misuse	9	2,079	15,837	2.40 (1.73, 3.31)	5.27	86
	Alcohol misuse: history	3	1,009	59,873	1.61 (1.31, 1.99)	4.45	19
	Drug misuse	8	1,262	91,208	1.96 (1.45, 2.64)	4.39	57
	Drug misuse: recent	4	254	2,320	1.80 (1.35, 2.38)	4.06	0
	Alcohol misuse	5	1,844	10,516	2.05 (1.29, 3.28)	3.01	68
Suicidality	Self-harm: history	7	1,164	125,758	1.85 (0.91, 3.79)	1.69	95
Treatment related	Medication: treatment adherence	4	261	2,575	0.59 (0.33, 1.06)	-1.77	80
	Medication: antipsychotic	3	990	59,702	0.42 (0.17, 1.01)	-1.94	94

[a] Recent: within last year

[b] PANSS: The Positive and Negative Syndrome Scale

[c] SES: Socioeconomic status

Table S8. Pooled odds ratios of risk factors for violence in psychosis when including only high quality studies

Risk factor domain	Risk factor[a,b]	K	No. Violent	No. Total	OR (95% CI)	Z-value	I^2
Criminal history	Violence history	7	1,969	84,978	3.81 (2.51, 5.77)	6.30	92
	Non-violent crime: history	4	932	21,151	6.17 (1.78, 21.37)	2.87	98
Premorbid	Victimization: violent, recent	3	135	36,496	4.94 (2.01, 12.14)	3.49	81
Psychopathological	Diagnosis: schizophrenia	3	64	880	1.68 (1.19, 2.36)	2.98	0
Sociodemographic	Education: no qualifications vs any	3	940	7,697	1.46 (1.26, 1.69)	5.00	4
	Sex: male	7	2,634	85,588	2.02 (1.28, 3.19)	3.03	95
	Marital status: single	5	917	17,787	1.35 (1.05, 1.72)	2.36	35
	Age: younger	4	983	59,827	1.63 (0.80, 3.32)	1.35	91
	Employment: unemployed	5	193	2,345	1.28 (0.86, 1.92)	1.22	45
	SES: low income	3	989	25,277	1.07 (0.52, 2.19)	0.17	96
Substance misuse	Alcohol misuse	3	407	10,244	2.10 (1.55, 2.84)	4.82	0
	Substance misuse	6	1,092	22,271	2.41 (1.68, 3.45)	4.79	84
	Drug misuse	5	2,124	75,918	2.17 (1.38, 3.42)	3.33	93
Suicidality	Self-harm: history	4	1,518	109,315	1.87 (1.06, 3.31)	2.16	94

[a] Recent: within last year

[b] SES: Socioeconomic status
